# A comprehensive characterization of rare mitochondrial DNA variants in neuroblastoma

**DOI:** 10.18632/oncotarget.10271

**Published:** 2016-06-24

**Authors:** Francesco Maria Calabrese, Rosanna Clima, Piero Pignataro, Vito Alessandro Lasorsa, Michael D. Hogarty, Aurora Castellano, Massimo Conte, Gian Paolo Tonini, Achille Iolascon, Giuseppe Gasparre, Mario Capasso

**Affiliations:** ^1^ Department of Biology, University of Bari “Aldo Moro”, Bari, Italy; ^2^ Department of Medical and Surgical Sciences-DIMEC, Medical Genetics Unit, University of Bologna, Bologna, Italy; ^3^ Department of Molecular Medicine and Medical Biotechnology, University of Naples Federico II, Naples, Italy; ^4^ CEINGE Biotecnologie Avanzate, Napoli, Italy; ^5^ Department of Pediatrics, Division of Oncology, Children's Hospital of Philadelphia, Perelman School of Medicine at The University of Pennsylvania, Philadelphia, USA; ^6^ Paediatric Haematology/Oncology Department, IRCCS, Ospedale Pediatrico Bambino Gesù, Rome, Italy; ^7^ Oncology Unit, IRCCS Istituto Giannina Gaslini, Genova, Italy; ^8^ Pediatric Research Institute (IRP) – Fondazione Città della Speranza, Neuroblastoma Laboratory, Padua, Italy; ^9^ IRCCS SDN, Istituto di Ricerca Diagnostica e Nucleare, Naples, Italy

**Keywords:** neuroblastoma, mitochondrial DNA mutations, WES, somatic mutations, germline mutations

## Abstract

**Background:**

Neuroblastoma, a tumor of the developing sympathetic nervous system, is a common childhood neoplasm that is often lethal. Mitochondrial DNA (mtDNA) mutations have been found in most tumors including neuroblastoma. We extracted mtDNA data from a cohort of neuroblastoma samples that had undergone Whole Exome Sequencing (WES) and also used snap-frozen samples in which mtDNA was entirely sequenced by Sanger technology. We next undertook the challenge of determining those mutations that are relevant to, or arisen during tumor development. The bioinformatics pipeline used to extract mitochondrial variants from matched tumor/blood samples was enriched by a set of filters inclusive of heteroplasmic fraction, nucleotide variability, and *in silico* prediction of pathogenicity.

**Results:**

Our *in silico* multistep workflow applied both on WES and Sanger-sequenced neuroblastoma samples, allowed us to identify a limited burden of somatic and germline mitochondrial mutations with a potential pathogenic impact.

**Conclusions:**

The few singleton germline and somatic mitochondrial mutations emerged, according to our *in silico* analysis, do not appear to impact on the development of neuroblastoma. Our findings are consistent with the hypothesis that most mitochondrial somatic mutations can be considered as ‘passengers’ and consequently have no discernible effect in this type of cancer.

## BACKGROUND

Neuroblastoma (NB) originates in tissues of the sympathetic nervous system, typically within the adrenal medulla or paraspinal sympathetic ganglia. It is the most common neoplasm diagnosed in infancy and accounts for 10–15% of childhood cancer mortality [1]. Clinical and genetic risk factors are used to stratify patients into prognostic subgroups that differ in the predicted biological aggressiveness of disease [2]. Unfortunately, those children with severe clinical course and widespread metastases, often with unfavorable tumor genetics, are categorized as ‘high risk’ and have survival rates < 40% despite aggressive and intensive therapies [3]. The genetic basis of neuroblastoma has recently come into focus, as multiple genomic loci highly associated with sporadic neuroblastoma by genome-wide association studies (GWASs) have been discovered [4–11]. In addition, the tyrosine kinase receptor *ALK*, the most frequently mutated gene in familial neuroblastoma [12], somatically acquired amplification of *MYCN* [13] and hemizygous deletions of chromosomes 1p and 11q [4] are highly recurrent and associated with poor prognosis [10]. We and other groups have recently investigated the recurrence of nuclear somatic mutations in neuroblastoma by using diverse next generation approaches [14, 15]. Surprisingly, neuroblastomas have shown an exceeding low somatic nuclear mutation rate beyond those in *ALK* and *ATRX* loci but there are several less frequently mutated genes with significant implications in cancer pathways [15–17].

Moreover, very recently, a recurrent rearrangement that activates telomerase in high-risk neuroblastoma was reported. To date, the screening of neuroblastoma genomic mutations has mostly focused on the investigation of nuclear DNA. Only recently a paper has investigated the mitochondrial DNA (mtDNA) variants in primary and relapsed tumor from patients with neuroblastoma, concluding that nuclear and mitochondrial variants showed a concordant increase during tumor progression but the mutations with functional impact were rare [18].

Indeed, mutations can frequently occur in mtDNA and may contribute to tumorigenic processes by modifying metabolic, apoptotic or hypoxic mechanisms [19, 20]. The identification of clearly deleterious mtDNA mutations in cancer tissues - such as an intragenic deletion [21] or the common tRNALeu (UUR) A3243G MELAS mutation, or, even more so, very rare clearly pathogenic mutations in complex I [22] (for a review see [19, 23]) - underline the relevance of pathogenic mtDNA mutations in neoplastic transformation. In recent years a high number of point mutations, either germline or somatic, insertions and deletions in mtDNA have been identified in multiple cancers [24–28]. Moreover, a recent worldwide effort by the mitochondrial community has resulted in the creation of a consortium named MSeqDR [29], with the aim to collect, integrate, organize and critically analyze mitochondrial sequence data. Consequently, nuclear clinical databases together with genomic browsers may be used as a mold for the mitochondrial counterpart.

Hence, in order to provide an extensive characterization of the occurrence of mtDNA mutations in neuroblastoma, two different approaches were adopted. First, the MToolBox pipeline [30] was used to extract the mtDNA sequences from a training set of 26 whole exome sequencing (WES) samples. Secondly, mtDNA from 33 fresh-frozen neuroblastoma specimens was entirely sequenced by Sanger technology. Hence, for the first time, a detailed and stringent workflow was applied to filter the numerous variants found to depict the neuroblastoma mtDNA mutational burden, which may potentially impact on the disease course. By using a multi-parametric workflow for the prioritization of mitochondrial DNA variants of clinical interest, we finally selected a mutation pool conceivably pathogenic [31, 32].

## RESULTS

### Mitochondrial DNA extraction, variant detection and annotation

**Figure 1:**
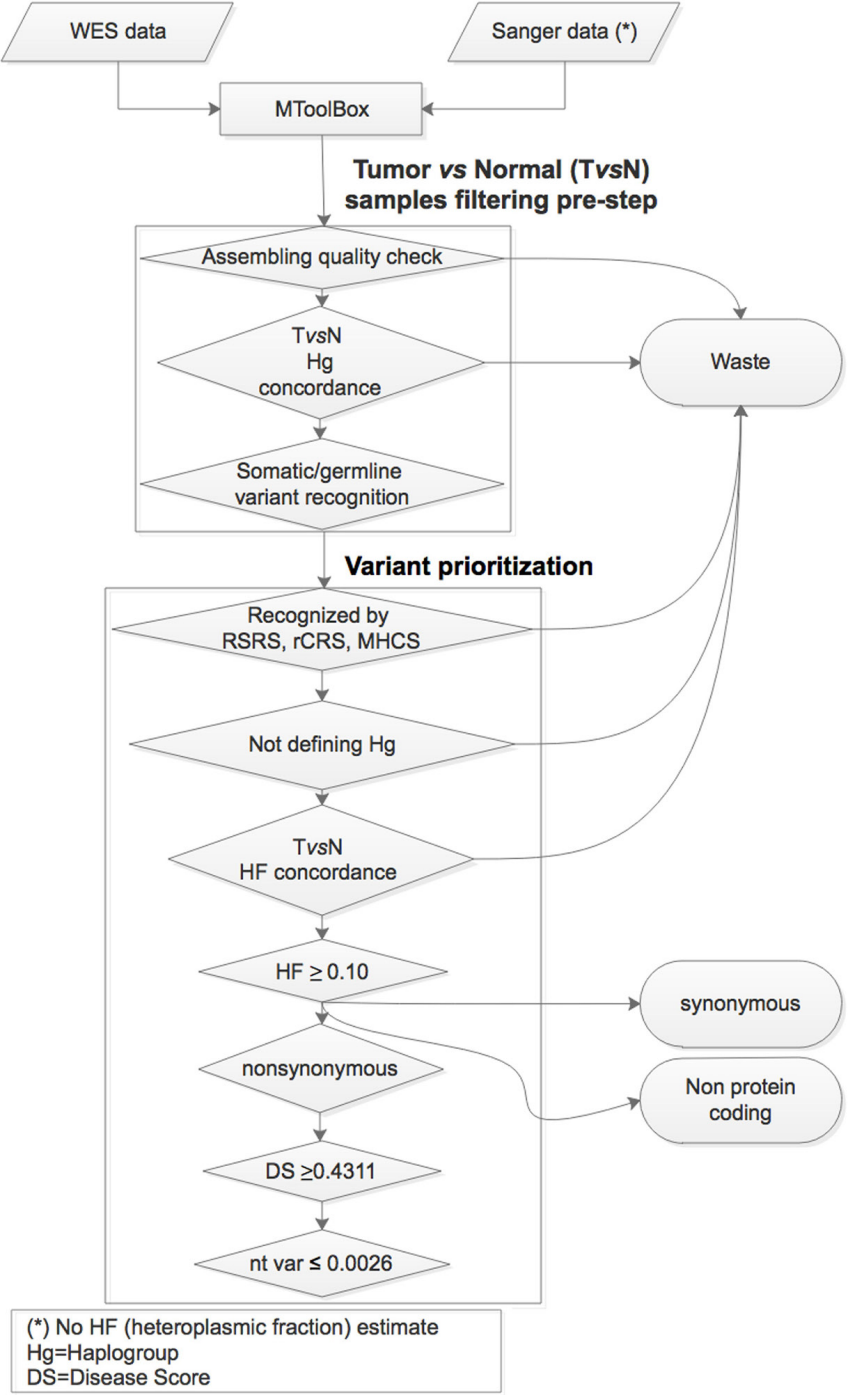
Variant analysis and prioritization workflow: the main steps useful to the variant analysis and prioritization phases are based on the implementation of MToolBox pipeline with various filters and cutoffs Disease score and nucleotide variability indexes are therefore accurately measured and evaluated. Finally, variants more likely contributing to defective phenotypes are selected.

The mitochondrial variant characterization here presented was based on the application of a multistep workflow (Figure 1). Next Generation Sequencing (NGS) technologies provide a robust high-throughput batch of data to be checked for comprehensive mutation detection. This ought to also include an accurate and sensitive measurement of heteroplasmy. Because we consider unwise to define as potentially pathogenic or likely damaging most of the heteroplasmic variants emerging from high coverage read depth sequencing, we adopted a multi-parametric workflow previously developed [32] based on an exhaustive functional annotation through the mtDNA extraction pipeline MToolBox and including i) Macro Haplogroup Consensus Sequences to filter out fixed evolutionary variants, ii) rare or private variants prioritization, iii) the nucleotide variability as reported in HmtDB and iv) the disease score, based on several predictors of pathogenicity for non-synonymous variants [30].

Mitochondrial mean coverage read depth and mitochondrial assembled bases in WES datasets ranged from 6.25X to 2184.92X and from 19.72% to 100%, respectively (Supplementary_Table_1). A phylogeny quality check of samples was performed using MToolBox for haplogroup assignment. The quality score of the haplogroup predictions ranged between 85.7% and 100% (Supplementary_Table_1). When somatic/germline mitochondrial haplogroup assignment did not match, exception made for phylogenetic closely related branches (subgroups or higher-level connected hierarchy branches), paired samples were not considered.

Overall, as reported in Supplementary_Table_1, the clinical phenotype in our datasets included 40 High Risk (HR), 18 Low Risk (LR) and 1 Intermediate Risk (IR) patient, according to the Children's Oncology Group (COG) classification system [2].

Because the mitochondrial assembled genomes and the depth of coverage for each samples were interconnected parameters useful in the quality sample estimate, in Supplementary Figure 1 the number of filtered mutations in both WES datasets was plotted against the relative mitochondrial mean of coverage depth in order to evaluate a potential correlation. Spearman's rank correlation coefficient (rho and linked *p*-value) showed a non-linear correlation between the two considered variables in each dataset (see note in Supplementary Figure 1). The overall correlations were not significant, indicating that an increase in the mean coverage did not correspond with an increased filtered single nucleotide polymorphism (SNP) number.

### Lack of association between specific mtDNA haplogroups and neuroblastoma

**Figure 2:**
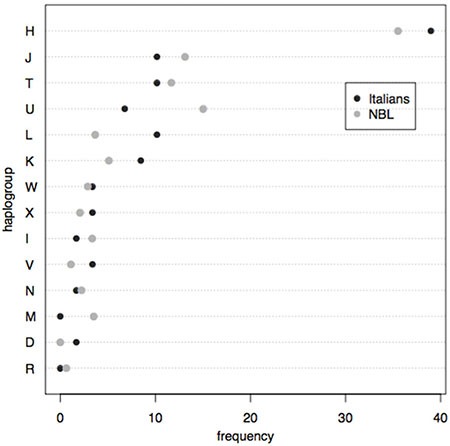
Macro-haplogroup frequency distribution in Italian neuroblastoma samples compared with the whole Italian healthy sample dataset retrieved from the HmtDB database shows the lack of association between specific mitochondrial haplogroups and disease occurrence

To investigate whether specific mtDNA haplogroups are associated to the occurrence of neuroblastoma, we compared the haplogroup frequencies observed in 43 Italian neuroblastoma samples within our cohort with those relative to 625 unrelated Italian healthy subjects, retrieved from the HmtDB public database [31] (Figure 2).

A binary logistic regression was performed on the entire datasets and no significant *p*-value resulted, suggesting that no haplogroup might be significantly associated with the occurrence of this cancer.

### WES germline variants

**Figure 3:**
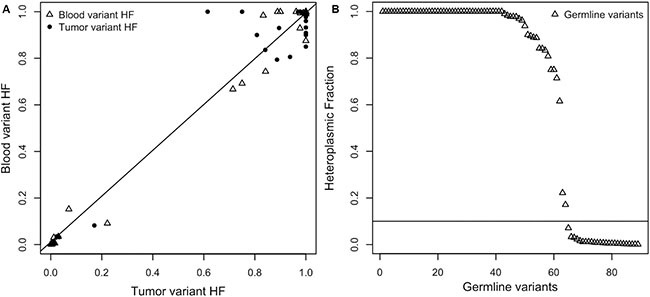
(**A**) Germline variant HF value correlation in blood and tumor. (**B**) The distribution of the heteroplasmic fractions (HFs) of germline variants shows how the majority of HFs (73%) had levels of heteroplasmy greater than 0.10.

Pairwise comparison of blood and tumor mtDNA variants was performed in order to identify mtDNA germline variants. In 25/26 samples we identified a total of 362 variants, 244 of which were found against the three mitochondrial references used. Within this filtered variant batch, 142 (58.2%) defined haplogroups and were not considered in further pathogenic prioritization steps. Thirteen out of 102 were non-haplogroup defining events and showed non-overlapping confidence intervals (CI) of heteroplasmic fraction (HF) in tumor/blood pairs. Thus, we focused our attention on 89 variants with matching CI of HF (Supplementary_Table_2, sheet 1). The scatter plot shows the correlation between the HF values of germline variants, across the whole set of WES samples (Pearson's product-moment correlation, *r* = 0.99, *p*-value = 2.2 × 10^−16^) (Figure 3A).

Sixty-four of the remaining 89 variants exhibited an HF value greater than the fixed threshold (0.10) which ranged from 0.171 to 1, as reported in Figure 3B and Supplementary_Table_2 (sheet 2).

Twenty-three variants were synonymous (Supplementary_Table_2, sheet 3), 16 were non-synonymous (Supplementary_Table_2, sheet 4) and 25 belonged to the non-protein coding class (Supplementary_Table_2, sheet 5).

According to the disease score fixed threshold, higher than 0.4311, 7 out of 16 non-synonymous variants resulted to be potentially pathogenic in 6 samples out of 25 (24%) (Table 1).

**Table 1:** List of germiline mutations identified by WES on neuroblastoma samples

Sample	Clinical phenotype	Variant Allele	HF Blood/ Tumor	Locus	Nt Var	Aa Change	Disease Score	Mitomap Associated Disease(s)
34C	HR	11361C	1/1	MT-ND4	9.60 × 10^−4^	M201T	0.709	
1579N	LR	14484C	1/1	MT-ND6	5.19 × 10^−3^	M64V	0.801	LHON
1C	HR	9055A	1/0.85	MT-ATP6	2.34 × 10^−1^	A177T	0.533	PD protective factor
24C	HR	11061G	0.99/0.995	MT-ND4	6.75 × 10^−3^	S101C	0.573	
8C	HR	14180A	0.962/1	MT-ND6	2.18 × 10^−2^	Y165F	0.522	
8C	HR	15665T	0.615/1	MT-CYB	0	L307F	0.770	
NB08N	HR	6419C	0.171/0.082	MT-CO1	0	K172N	0.782	

Abbreviations: LR: Low Risk; HR: High Risk; HF: Heteroplasmic Fraction; Nt Var: nucleotide variability; LHON: Leber Hereditary Optic Neuropathy; PD: Parkinson Disease.

All reported non-synonymous mutations were detected as homoplasmic or nearly-homoplasmic in both blood and tumor, with the exception of the mutation m.6419A > C/*MT-CO1* which exhibited a HF value close to the fixed threshold of 0.10. Although more than half of all samples harbored non-synonymous mutations in complex I gene subunits, the only one belonging to a low risk clinical phenotype (Italian sample 1579N) carried the deleterious homoplasmic mutation m.14484T > C (MT-ND6 Met64Val) also reported as associated to disease in Mitomap [33]. This mutation, strongly expected to predispose to a specific mitochondrial disease, was extensively published as one of the three most common mutations associated with Leber Hereditary Optic Neuropathy and responsible to induce a subtle reduction of complex I activity [19]. Moreover, the mutation m.9055G > A, found within the *ATP6* gene in the sample 1C, was described to decrease Parkinson Disease risk [34]. All reported non-synonymous mutations had nucleotide variability ranging between 0 and 0.23. When we applied the nucleotide variability cutoff 0.0026 [32, 35], only three variants (m.11361T > C/*MT-ND4*, m.15665C > T/*MT-CYB*, m.6419A > C/*MT-CO1*) were classified as potentially pathogenic.

Among the 25 non protein-coding events, 20 mapped to the *MT-DLOOP* region of 15 individuals (Supplementary_Table_2, sheet 5). These batches included variants annotated as polymorphisms in Mitomap, as confirmed by their high nucleotide variability score and the reported allele frequency in the 1000 Genomes database. All 20 *MT-DLOOP* variants were homoplasmic (or nearly homoplasmic) and all defined a different haplogroup than the assigned one. Among non-protein coding variants, two resided within the rRNA12S locus of the same sample and three in tRNA loci. The secondary structure of mutated rRNA12S showed no modification and a nearly unvaried minimum free energy (data not shown). Three variants belonged to tRNA loci, namely the m.14687A > G/*MT-TE*, the m.15946C > T/*MT-TT* and the m.10463T > C/*MT-TR*. PhyloP and PhastCons tRNA conservation scores, consulted to check variant position preservation during evolution and functional constraints, were equal to 1.56 and 0.82, −0.98 and 0, −1.55 and 0 in glutamic acid, threonine and arginine tRNAs, respectively. These different values pointed out how the position m.14687 of tRNA glutamic acid resulted to be phylogenetically more conserved than the other two. All germline tRNA variants contributed to define other haplogroups, somewhat diminishing the possibility of a pathogenetic role.

Briefly, although the germline defined variants were overall 362, no one of these appeared to be potentially pathogenic within the non-protein-coding subset; only 7 (2%) complied with all prioritization criteria, mapped in protein-coding genes, and were therefore selected as potentially pathogenic mutations (Table 1).

### WES somatic variants

**Figure 4:**
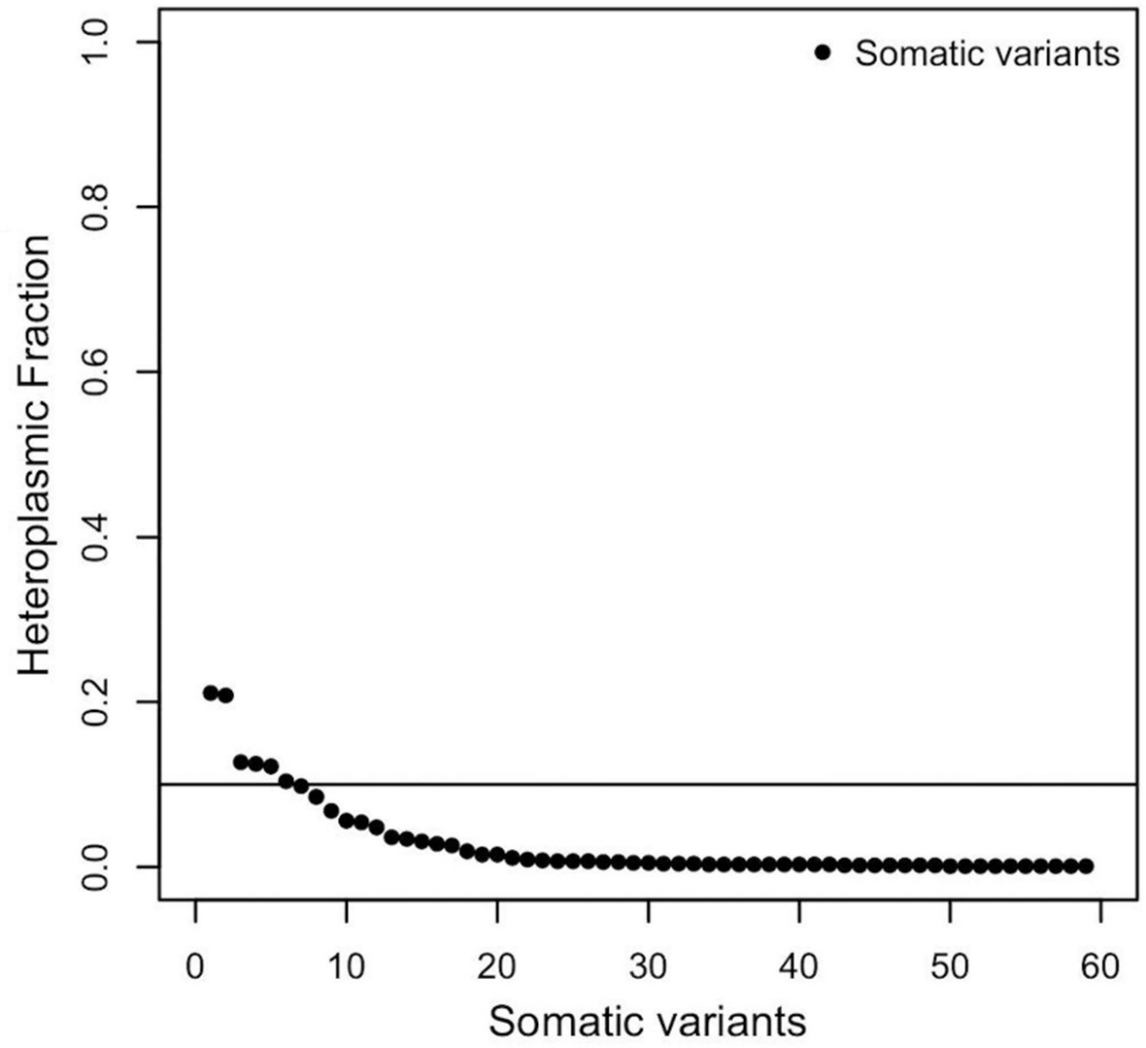
Distribution of the heteroplasmic fractions (HFs) of somatic variants

Those events uniquely detected in tumor samples were annotated as somatic variants. To assess this status, a further check was performed on each predicted germline paired variant, looking for those sites not detectable due to a coverage lower than the minimum of 5 in MToolBox. We identified 119 false positive somatic mutations (Supplementary_Table_3, sheet 1). The analysis was thus centered on a total of 60 somatic variants, all recognized with respect to the three mitochondrial references used (Supplementary_Table_3, sheet 2). Of these, 59 variants did not contribute to define the sample haplogroup (Supplementary_Table_3, sheet 3). The majority of them (89.8%) showed a HF lower than 0.10 and were consequently discarded (Figure 4). The remaining 6 variants had low HF (0.104 – 0.211) and the nucleotide variability did not exceed the value of 0.011 (Supplementary_Table_3, sheet 4); one was synonymous (16.7%) (m.8239C > T*/MT-CO2*), and five belonged to the non-synonymous variant class (Supplementary_Table_3, sheet 5).

Among the non-synonymous variants, 4 were recognized as potentially pathogenic (Table 2) in as many different samples. The mutation m.11090A > C/*MT-ND4* with a disease score of 0.85 co-occurred in two samples (2221T and 1940T). The 34S sample had a mutation in the *MT-CO3* gene with the highest HF value (0.208) and a relative null nucleotide variability value (Table 2). All potentially deleterious WES somatic mutations did not contribute to define other haplogroups and were not annotated in Mitomap or OMIM in association to disease.

**Table 2:** List of somatic mutations identified by WES on neuroblastoma samples

Sample	Clinical phenotype	Variant Allele	HF	Locus	Nt Var	Aa change	Disease Score
34S	HR	9625T	0.208	MT-CO3	0	S140L	0.857
NB11PT	HR	10306C	0.125	MT-ND3	0	N83T	0.503
2221T	HR	11090C	0.122	MT-ND4	5.10 × 10^−4^	T111P	0.850
1940T	HR	11090C	0.104	MT-ND4	5.10 × 10^−4^	T111P	0.850

Abbreviations: HR: High Risk; HF: Heteroplasmic Fraction; Nt Var: nucleotide variability.

Overall, all non-synonymous variants showed nucleotide variability and disease score respectively lower and higher than the fixed thresholds.

### Mitochondrial DNA mutations in snap-frozen neuroblastoma samples

Full mtDNA sequencing was also performed by Sanger technology in 33 snap-frozen tumor biopsies (Supplementary_Table_1). The corresponding blood-extracted DNA allowed us to estimate the germline or somatic nature of potentially pathogenic mutations in our set. A total of 476 mtDNA events were identified (Supplementary_Table_4, sheet 1). Of this first batch, 287 were recognized by all the three mitochondrial references (Supplementary_Table_4, sheet 2). The variants contributing to haplogroup assignment were 191 and were discarded from the subsequent analyses. The analysis was centered on 96 non-haplogroup defining variants (Supplementary_Table_4, sheet 3), 78 of which mapped within protein-coding regions (Supplementary_Table_4, sheet 4) and 18 in rRNAs and *MT-DLOOP* loci (Supplementary_Table_4, sheet 5). The non-protein-coding variants displayed variability values equal to or lower than 0.031. Specifically, 2 resided within the regulatory *MT-DLOOP* region and were not previously reported in Mitomap. Moreover, 7 patients harbored variants in tRNA genes. Four did not define other haplogroups and had the lowest nucleotide variability score (Table 3). When we looked at the tRNA secondary structure, we noticed that the somatic m.7506G > A variant in the serine tRNA (*MT-TS1*) locus mapped in the D arm stem and was previously reported as associated with progressive external ophthalmoplegia and hearing loss, in heteroplasmy [36] (Table 3). Two germline variants mapped on tRNA-Thr (*MT-TT*) both in the stem of the T arm: the first m.15943T > C did not impact on secondary structure, the second one (m.15944T > C) is annotated in MAMIT-tRNA [37] as a polymorphism (Table 3). We found the germline m.10023C > G/*MT-TG* mutation, whose position is in a highly evolutionarily conserved site, i.e. the anticodon stem-loop (Table 3). The m.10023C > G mutation would cause the carrying of the amino acid arginine instead of glycine. The two amino acids belong to different chemical classes; the non-polar charged glycine would therefore be substituted with a hydrophilic polar charged one. We therefore deemed this one mutation to be the only one potentially interesting from the clinical point of view, although the lack of data on the segregation of the same variant in the individual's mother does not allow us to conclude on a potential predisposing effect on neuroblastoma of this mutation.

**Table 3:** List of mutations identified in tRNAs by Sanger sequencing on snap-frozen neuroblastoma samples

Sample	Clinical phenotype	Variant Allele	Status	HF^1^	Locus	Nt Var	Mitomap Associated Disease(s)
1360	HR	10023G	germline		MT-TG	0	
1506	LR	7506A	somatic		MT-TS1	0	PEO with hearing loss
1493	HR	15943C	germline		MT-TT	1.0 × 10^−3^	
1360	HR	15944C	germline		MT-TT	2.0 × 10^−3^	

Abbreviations; HR: High Risk; LR: Low Risk; Nt Var: nucleotide variability; PEO: Progressive External Ophthalmoplegia.

1 Heteroplasmic fraction is not available for mutations identified with Sanger sequencing. Here only heteroplasmic (+) vs. homoplasmic (−) status is reported.

Ribosomal RNA genes were also found to harbor 7 substitutions. Four of them were in the *MT-RNR2* and 3 in the *MT-RNR1* gene, in seven different samples (Supplementary_Table_4, sheet 5). Prediction of the secondary structure of the rRNA mutated molecules showed no evident structural changes for all substitutions compared to the wild-type conformation (data not shown).

Twenty-six non-synonymous variants (Supplementary_Table_4; sheet 6) were detected in 17 out of 33 samples (51.5%), 10 of which were predicted to impair protein function by a disease score higher than 0.4311. This number was further reduced to 6 potentially pathogenic variants, when the nucleotide variability cutoff filter was applied (Supplementary_Table_4, sheet 7). All of these considered variants were exclusive to each sample. In addition, one frame-shift event was also detected (Supplementary_Table_4, sheet 7).

Upon assessing the germline/somatic status of 7 of these potential pathogenic mutations in protein coding regions, we found 2 germline and 5 somatic mutations (Table 4).

**Table 4:** List of prioritized mutations identified by Sanger sequencing on snap-frozen neuroblastoma samples

Sample	Clinical phenotype	Variant Allele	Status	HF^1^	Locus	Nt Var	AA Change	Disease Score
1332	HR	15284G	germline	−	MT-CYB	0	T180A	0.626
1360	HR	12014T	germiline	+	MT-ND4	2.0 × 10^−3^	L419F	0.524
1090	LR	7402A	somatic	−	MT-CO1	0	P500Q	0.748
1506	LR	8441A	somatic	−	MT-ATP8	1.0 × 10^−3^	L26M	0.784
		9056A	somatic	−	MT-ATP6	1.0 × 10^−3^	A177D	0.729
1047	HR	14945A	somatic	−	MT-CYB	1.0 × 10^−3^	A67T	0.678
1144	HR	11915d	somatic	−	MT-ND4	0	frameshift	

Abbreviations: HR: High Risk; HF: Heteroplasmic Fraction; Nt Var: nucleotide variability.

1 Heteroplasmic fraction is not available for mutations identified with Sanger sequencing. Here only heteroplasmic (+) vs. homoplasmic (−) status is reported.

None of the germline and somatic mutations were found to be associated to disease in Mitomap or OMIM. Sample 1360 harbored the only one mutation found in heteroplasmy (C/T) in the blood (m.12014C > T/*MT-ND4*). The remaining 2 germline mutations mapped in the gene *MT-ND4* encoding for a complex I subunit (Table 4).

All 5 reported somatic mutations had a high disease score ranging between 0.678 and 0.784 (Table 4). One frameshift mutation occurring in the *MT-ND4* gene was detected (Table 4).

## DISCUSSION

In this study, we intended to ascertain whether mitochondrial DNA variants might be linked to neuroblastoma. The MToolBox pipeline and a multi-parametric workflow, both recently developed [30, 32], were used to investigate and prioritize the germline and somatic mutations in two WES datasets of tumors and blood pairs of 26 individuals. Moreover, full mtDNA from 33 snap-frozen neuroblastoma samples was sequenced with Sanger method and analyzed with MToolBox as well. A similar strategy was previously exploited in an analogous fashion by our group on glioblastoma multiforme [38]. The identification and the understanding of genetic events that drive cancer is a crucial phase to improve diagnostic, prognostic and therapeutic strategies. Hereby, we here intended to explore the mitochondrial variation and its possible involvement in neuroblastoma.

Neuroblastoma, like most other solid tumors, shows a high glucose uptake [39, 40], together with a high rate of lactic acid production and a low rate of oxygen consumption, reflecting the switch from mitochondrial OXPHOS to glycolysis [41]. Moreover, a down-regulation of all components of the aerobic mitochondrial energy metabolism without affecting mitochondrial mass was recently observed [42]. Recently, an *in vitro* study on the neuroblastoma cell lines LA-N-1, IMR-32, LS and SK-N-SH, showing an increased oxidative stress, a reduced lactate dehydrogenase (LDH) enzyme activity and reduced lactate production after the use of the antiprotozoal drug nifurtimox, supports the previous finding [43]. Moreover, it is becoming clearer how many oncogenic key signaling pathways converge to adapt tumor cell metabolism in order to support cancer growth and survival. Indeed, the glycolytic shift in the presence of oxygen (Warburg effect) has been inferred in many cancers, including neuroblastoma [39, 41, 44]. In the last decade several groups have investigated changes of the OXPHOS system in cancer. It has been proven that, depending on the tumor type, cancer cells are frequently characterized by either a generally low amount of mitochondria and OXPHOS [45], a specific defect of one OXPHOS complex [46–49] or a combined low level of OXPHOS complexes [50]. There are strong evidences that the Warburg effect in neuroblastoma is not caused by a low mitochondrial mass per cell [51]. Thus, defects of mitochondrial functions may be relevant modifiers events in neuroblastoma, from which derived our need to investigate whether this cancer type may represent a paradigm in which the Warburg effect is due to mtDNA mutations. Nonetheless, our data do not point to a major involvement of mtDNA mutations in neuroblastoma genesis or progression, as the burden of the genetic lesions that pass stringent prioritization filters for a potential pathogenic role was limited.

Overall, we found very few somatic mutations, in agreement with recent high-throughput screenings of nuclear DNAs which have demonstrated the relative paucity of recurrent somatic mutations in neuroblastoma and highlighted the difficulty to develop therapeutic strategies, relying on frequently altered oncogenic drivers [14, 15].

Specifically for mtDNA, one potential explanation for this is that these tumors arise in very young individuals, whereas accumulation of mtDNA mutations is a phenomenon typical of aging tissues. It is also true, however, that it is becoming increasingly evident that most mtDNA mutations, particularly if detected in low heteroplasmies, may result from intrinsic mtDNA replication mechanisms and are to be considered passengers events during tumorigenesis, few of which only becoming fixed in the quickly-evolving cancer cell population [52]. With respect to the few potentially pathogenic mutations found, it is worth to mention that, although aware of the low statistical power to attempt a correlation, we found no association of the former with *MYCN* ploidy, based on the rationale that the proto-oncogene *MYCN* is indirectly involved in regulating the switch off of mitochondrial function via a positive regulation of aerobic glycolysis and conversion of pyruvate to lactate [43, 53]. Its amplification, therefore, besides being associated to a poorer prognosis in neuroblastoma, may favor a relaxed selection of mtDNA variants. This does not appear to be the case, based on the limited data we gathered in our cohorts, albeit further studies are warranted on this issue.

Overall, apart from the environmental factors acting over mtDNA mutations which may easily concur to this multifactorial neoplasm, our *in silico* data, when merged with the other evidences present in the scientific literature on the metabolic features of neuroblastoma, suggest a mechanism of OXPHOS reduction likely independent from the occurrence of mtDNA mutations.

## MATERIALS AND METHODS

### Ethics statement

The present study was formally conducted on two different datasets, one composed of Italian and one of American neuroblastoma patients. Data relative to the first one, obtained both with Sanger and next generation sequencing (NGS) technology, were approved by the Ethics Committee of the Medical University of Naples; the written informed consent was obtained by all children's legal guardians. The other batch of analyzed WES, stored in the European Genome-phenome Archive (EGA) database, was used with the approval obtained from The Children's Hospital of Philadelphia Institutional Review Board. This second dataset is part of an enlarged neuroblastoma study published by Sausen in 2013 [54].

### WES Children's Oncology Group (COG) samples

Patient details and any associated reference for the 16 matched primary tumor/normal samples belonging to the COG clinical trials cooperative group biobank here used, are retrievable in the online version of the previously cited paper (REF) and resulted as part of the EGA project entry registered under Acc. No EGAS00001000369.

### WES and snap-frozen DNA neuroblastoma samples

In-house WES data of neuroblastoma samples were used to retrieve the mtDNA sequences. DNA samples for Sanger sequencing were obtained from the BioBank at IRCCS AOU San Martino-IST. Agilent SureSelect Target Enrichment System 50 Mb (Agilent Technologies, Santa Clara, CA) was used for exome capture (10 normal/tumor DNA) according to the manufacturers' protocol. Captured DNAs were subjected to massively parallel sequencing using Illumina HiSeq 2000 (Illumina Inc., San Diego, CA) with 100 bp paired-end reads, while 33 DNA samples were sequenced by using Sanger sequencing technology.

### mtDNA amplification and sequencing (sanger method)

Single PCR volumes were optimized in order to allow a better performance amplification on mtDNA and were set as follows: 25 μl in order to allow a better performance amplification (HiFi HotStart DNA Polymerase - KAPABIOSYSTEMS), 3.5 μl primer forward + reverse each one concentrated 10 μM, 6 μl DNA (30 ng tot.) and 3 μl H_2_O. 46 overlapping amplicons in order to cover full-length mtDNA (available upon request) were used. Cycle amplification were made of 3′ to 95°C, 15ʺ to 95°C, 5ʺ to 60°C and 5ʺ to 72°C (× 40 times), finally 10′ to 72°C. The amplified amplicons were purified and sequenced by using a 3730 DNA Analyzer (Applied Biosystem).

### Haplogroup frequency statistics

A set of 625 Italian healthy samples retrieved from the human mitochondrial public database HmtDB [31] was used as haplogroup control group and was tested against our Italian neuroblastoma dataset.

Statistical analyses were performed using R environment, version 3.1.2, and statistical significance was established at *p*-value ≤ 0.05. Binary logistic-regression analysis was used. In order to test a potential haplogroup contribution to neuroblastoma etiology or predisposition, a logistic regression of all categories at once was performed. We analyzed the haplogroup categories in patients and controls using the common haplogroup H as the reference level. Spearman rank test was used to assess the correlation between mitochondrial mean coverage read depth and the number of filtered variants. Pearson product-moment correlation coefficient, a measure of the strength and direction of association that exists between two continuous variables, was applied.

### Retrieving of mtDNA reads and prioritization criteria

All WES dataset fastq and fasta Sanger sequence files were analyzed by using the MToolBox pipeline [30], which helps filtering and prioritizing pathogenic variants.

For each tumor/blood samples, three parameter discrepancies resulting from MtoolBox, i.e. mitochondrial read depth, assembled bases and haplogroup assignment were considered; if these values for a tumor/normal pair were not comparable, samples were discarded from the analysis. Moreover, because the read depth threshold required by MToolBox filtered out variants with a depth lower than 5×, the germline counterpart in the mtDNAassembly-table output was checked, in order to manually assess, by inspecting the coverage, the somatic status for each detected variant. Further, we considered as potentially pathogenic variants only those recognized against the mitochondrial reference sequences (revised Cambridge Reference Sequence (rCRS), Recostructed Sapiens Reference Sequence (RSRS) and Macro Haplogroup Consensus Sequence (MHCS)) which occurred in non haplogroup-defining sites, which featured a heteroplasmy fraction greater than 0.10, featuring nucleotide variability lower than nucleotide variability cutoff (0.0026) and having a disease score above the disease score threshold (0.4311). We also checked the Heteroplasmy Fraction (HF) confidence interval related concordance in variants shared between tumor/normal-paired samples. For the whole set of selected variants the nucleotide variability value, both for somatic and germline samples, was estimated [35]. Intuitively, mitochondrial low variability values, directly linked to positions not prone to vary, were associated more likely to potentially pathogenic mutations [55]. To evaluate if the number of mitochondrial variant positions (found versus all the three reference sequences and singularly – without any other applied filter) and *MYCN* amplification status correlated, a Wilcoxon test (R environment) was performed.

The evaluation of tRNA variants was based on the phastCons score [56], which takes into account neighboring bases and reports the probability that each nucleotide belongs to a conserved element, and on the phyloP algorithm [57], useful in evaluating signatures of selection at particular nucleotides, ignoring neighboring bases in its calculation and producing a *p*-value. The second algorithm measures both acceleration (faster evolution than expected under neutral drift) and conservation (slower than expected evolution).

The prediction of RNA secondary structures was performed by RNA Folding Form (version 2.3 energies) software, using the set of default parameters [58].

### Heteroplasmy fraction threshold

Because of the uncertain potential degree of contamination affecting the tumor tissue, which is not measurable by analyzing the non-tumor counterpart, a heteroplasmy underestimation ought here to be considered. Further, another limitation that may affect the mitochondrial coverage and therefore the prioritization process, deals with the enrichment technology used. As noticed in human exome projects, different enrichment technologies showed different off-target read distributions [59]. The highest mitochondrial read coverage distributions were observed in association with lower density platforms. Both the WES sequencing used in this paper are based on SureSelect Agilent enrichment technology which showed an intermediately mitochondrial coverage read depth distribution together with a corresponding reconstructed genome percentage in terms of reconstructed contigs. The SuresSelect ranks between other two powerful used platforms: the Illumina TrueSeq and Nimblegen SeqCap EZ-exome platform. Thus, after considering both the uncertain level of tumor tissue contamination and the off-target mitochondrial spectrum platform dependence, we forthwith set the HF threshold to 10%. A similar HF threshold was used for other heterogeneous tumor like glioblastoma [38].

## CONCLUSIONS

Although a high number of mitochondrial variants was detected, the large majority of them did not pass the prioritization filters, as the WES detecting capability can be considered a double edge sword, which sums up a huge number of mutations which we opportunely filtered in order to recognize those of potential clinical interest. Therefore, a relatively limited burden of pathogenic mutations is indeed carried by neuroblastoma suggesting that they may be passenger events rather than potent modifiers in cancer progression in this cancer type. More efforts are warranted to understand if mtDNA contributes at all to neuroblastoma aggressive behavior, its progression and its metabolic plasticity.

## SUPPLEMENTARY MATERIALS FIGURES AND TABLES










