# Supplementary material for: A comprehensive characterization of rare mitochondrial DNA variants in neuroblastoma
Source: Oncotarget. 2016 Jun 24;7(31):49246–58. doi: 10.18632/oncotarget.10271 (PMC5226504; doi:10.18632/oncotarget.10271)
Supplement: Supplementary file 1 [file oncotarget-07-49246-s001.pdf]

A comprehensive characterization of rare mitochondrial DNA variants in neuroblastoma

Supplementary Materials

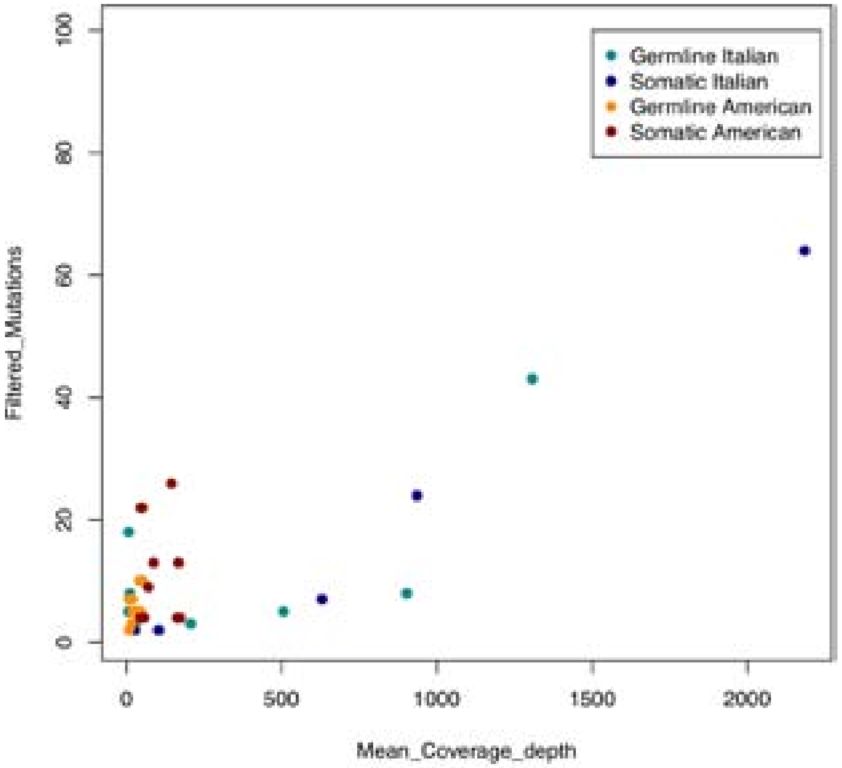

	rho	*p-value*
Italian Germline WES	0.1772294	0.6483
Italian Somatic WES	0.9746794	0.004818
American Germline WES	0.4690062	0.09069
American Somatic WES	0.0087435	0.9822

Supplementary Figure S1: (**A**) Correlation scatterplot between mean coverage read depth and number of filtered mutations in all considered WES dataset (**B**) Mean coverage read depth and filtered number of varients have been correlated using the spearman rank statistic correlation. Among the four considered datasets, only the one which gathers the italian somantic varients showed a significant correlation (rho value), probably due to the small sample size.

Supplementary Table S1: Clinical phenotype sample data together with mitochondrial sequencing statistics.

Supplementary Table S2: Total variant class results. Sheet 1 – total variants with matching CI of HF; sheet 2 – variants exhibiting an HF value greater than the fixed threshold; sheet 3 – synonymous variants; sheet 4 – non-synonymous variants, sheet 5 – non-protein coding class variants

Supplementary Table S3: WES somatic variant class results. Sheet 1 – WES false positive somatic mutations; sheet 2 – WES somatic variants recognized against the three mitochondrial references; sheet 3 – non-defining haplogroup WES somatic variants; sheet 4 – WES somatic variants exhibiting nucleotide variability lower than 0.011; sheet 5 – WES somatic synonymous and non-synonymous variants under the fixed threshold.

Supplementary Table S4: Snap-frozen sequencing variant class results. Sheet 1 – total Sanger identified variants; sheet 2 – Sanger variants recognized against the three mitochondrial references; sheet 3 – non-haplogroup defining Sanger variants; sheet 4 – Sanger variants mapping within protein-coding regions; sheet 5 – sanger variants in rRNAs and *MT-DLOOP* loci; sheet 6 – non-synonymous Sanger variants; sheet 7 – potentially pathogenic Sanger variants after applying the nucleotide variability cutoff filter.
